# Continuous Anaerobic Digestion of Wood Vinegar Wastewater From Pyrolysis: Microbial Diversity and Functional Genes Prediction

**DOI:** 10.3389/fbioe.2020.00923

**Published:** 2020-08-05

**Authors:** Dongliang Hua, Qingwen Fan, Yuxiao Zhao, Haipeng Xu, Lei Chen, Hongyu Si, Yan Li

**Affiliations:** ^1^Shandong Provincial Key Laboratory of Biomass Gasification Technology, Shandong Academy of Sciences, Energy Research Institute, Qilu University of Technology, Jinan, China; ^2^State Key Laboratory of Microbial Technology, Shandong University, Qingdao, China

**Keywords:** anaerobic digestion, bacterial community, functional genes, methanogenesis, wood vinegar wastewater

## Abstract

Wood vinegar wastewater (WVWW) is the main by-product of biomass pyrolysis process, which is more suitable to use anaerobic digestion (AD) to achieve energy recovery due to its large amount of organic matter. In this study, the up-flow anaerobic sludge bed (UASB) reactor was used to investigate the continuous anaerobic transformation of WVWW with gradient concentrations (0.3, 0.675, 1, 2, 3, 4, 5, 6, and 7 g COD/L). Then, the changes of microbial community, diversity index and functional gene were analyzed in detail. The results revealed that WVWW showed good AD performance in continuous fermentation. WVWW of organic loading rate (OLR) of >8.58 g COD/L⋅d showed severe inhibition on biodegradability and methane production, which is mainly due to the toxic substances as compared with the control group. The bacterial communities were dominated by phyla of *Chloroflexi*, *Firmicutes*, *Proteobacteria*, *Acidobacteria*, *Synergistetes*, and *Actinobacteria*. The gene abundances related to energy production, carbohydrate transport and metabolism were relatively high, which are mainly responsible for carbon forms conversion and carbohydrate degradation. This study will provide a basis for the screening and enrichment of functional bacteria and genes.

## Introduction

Pyrolysis of biomass is currently the most attractive and prospective technology for converting biomass into renewable energy such as bio-oil and biochar (Zhu et al., 2019; Shen et al., 2020). Nevertheless, improper disposal of the pyrolysis by-product, wood vinegar wastewater (WVWW), causes environmental pollution and waste of resources, considering its high organic concentration and complex composition (Zhang et al., 2019a). Anaerobic digestion (AD) is a preferred method for adequately disposing organic waste because it involves high controllability, low operating cost, and production of methane with low carbon footprint. WVWW is a high-strength organic wastewater characterized by high chemical oxygen demand (COD, 110–120 g/L) (Zhang et al., 2019b), with acetic acid commonly accounting for about 30–70% of the total organic compounds (Theapparat et al., 2015). Since acetic acid is an essential precursor of anaerobic methanogenesis, AD of WVWW is justified.

According to (Pimenta et al., 2018) and Si et al. (2018) the organic characteristics of WVWW are similar to those of hydrothermal liquefaction wastewater (HTLWW), suggesting comparable anaerobic digestibility. Currently, the biological treatment of WVWW mainly involves aerobic technology, serving as an additive for investigating the effect of aerobic microbial activity and competitive adsorption of heavy metals (Liu et al., 2018; Zhang et al., 2019a, b), whereas HTLWW is primarily degraded by AD for recovering energy. In addition to small biodegradable organic molecules (organic acids, alcohols, etc.) HTLWW also contains refractory molecules including phenols and N/O heterocyclic compounds which inhibit the methanogenic activity of anaerobic microorganisms (Wirth et al., 2015; Zheng et al., 2016). Tommaso et al. (2015) studied the anaerobic digestibility of HTLWW, concluding that the wastewater produced at high temperatures exhibited better cumulative methane production and anaerobic performance. Nevertheless, the high concentration (≥13.3%) of HTLWW, severely inhibits anaerobic microbial activity (Zheng et al., 2016; Fan et al., 2020). Diverse methods have been employed to improve the anaerobic methanogenic potential of HTLWW including physical absorption, photocatalytic oxidation, and ozonation. Several studies have also revealed that physical adsorbents such as activated carbon and zeolite increase the cumulative methane yield and mitigate inhibition by toxicants (Zheng et al., 2016; Usman et al., 2019). Photocatalyzed HTLWW can enhance methane production and the COD removal rate, increasing the total energy recovery to 66.7% (Quispe-Arpasi et al., 2018). Yang et al. (2018) utilized ozonation to efficiently convert phenols and N-heterocyclic compounds in HTLWW, with BOD_5_/COD increasing by 32.36% (maximum of 0.41). These studies demonstrated that AD is a feasible method for degrading HTLWW that can significantly improve the energy recovery of the hydrothermal liquefaction process system (Gu et al., 2019). Additionally, the microbial community structures were directly related to the degradation efficiency of the organic wastewater. Microbial community analysis revealed high amounts of bacteria capable of degrading N-heterocyclic and aromatic compounds such as *Syntrophorhabdus* and *Synergistes* (Usman et al., 2019). Si et al. (2016) also reported numerous microorganisms associated with acidification and detoxification in long-term continuous AD experiments.

In the current study, we focus on the anaerobic digestibility of WVWW with gradient organic lading rates (OLRs). The daily methane production and biodegradability (BD) are measured to evaluate continuous methanogenesis of the gradient OLRs. Since the microbial community exerts influence on organic wastewater degradation, the structures and diversity of the community under diverse OLRs are analyzed by high-throughput sequencing technology. The aim is to elucidate the function of and abundance of different genes, suitable for enriching detoxifying bacteria and screening of functional genes.

## Materials and Methods

### Materials

The WVWW was produced by condensation of steam from peanut shell pyrolysis at 500°C, and the physicochemical characteristics involved a COD concentration of 110 g/L, ammonia concentration of 45.3 g/L, and pH of 3–4. The WVWW was stored at 4°C. And granular sludge containing 9.74% total solids (TS, % w/w) and 75.35% volatile solids (VS, % of TS) was collected from up-flow anaerobic sludge bed (UASB) reactor treating sugar processing wastewater of the organic wastewater treatment plant in Shandong, China. It was continuously fed with 1 g COD/L sodium acetate in UASB reactor for 30 days to maintain microbial activity. All chemical reagents used in the experiments were of analytical grade.

### Experimental Design

The UASB reactor was used to culture anaerobic microorganisms and enrich functional genes with increasing OLRs. The effective volume of the 7 cm diameter and 41 cm high reactor was 2.8 L. Half of its effective volume was filled with granular sludge and maintained at 38°C by constant temperature circulating water, and the hydraulic retention time (HRT) was set at 48 h through a peristaltic pump (BT100-2J, LONGER, China). Samples of WVWW were prepared by diluting to 0.3, 0.675, 1, 2, 3, 4, 5, 6, and 7 g COD/L, with corresponding OLRs of 0.43, 0.97, 1.43, 2.86, 4.29, 5.72, 7.15, 8.58, and 10.01 g COD/L⋅d. The pH of the influent was adjusted to approximately 7.0 using 3 mol/L NaOH and trace elements essential for microbial metabolism were added to retain 1 mL/g COD. The components and concentrations were as follows: H_3_BO_3_ (50 mg/L), NiCl_2_⋅6H_2_O (50 mg/L), MnCl_2_⋅4H_2_O (50 mg/L), (NH_4_)_6_Mo_7_O_24_⋅4H_2_O (50 mg/L), AlCl_3_⋅6H_2_O (50 mg/L), CuSO_4_⋅5H_2_O (80 mg/L), ZnSO_4_⋅7H_2_O (80 mg/L), CoCl_2_⋅6H_2_O (200 mg/L), MgCl_2_ (4000 mg/L), and CaCl_2_ (4000 mg/L). A control experiment was conducted simultaneously using sodium acetate as the substrate, while other operating procedures were similar to that for WVWW samples. Each OLR stage lasted 15 days, with the gas volume generated and its composition measured. The granular sludge was sampled from the bottom of the UASB reactor for high-throughput sequencing analysis at the end of OLRs for the 1.43, 2.86, 4.29, 5.72, 7.15, 8.58, and 10.01 g COD/L⋅d experiments, corresponding to influent organic concentrations of 1, 2, 3, 4, 5, 6, and 7 g COD/L. The microbial samples were labeled as IC-1, IC-2, IC-3, IC-4, IC-5, IC-6, and IC-7. At the end of the 10.01 g COD/L OLR experiment, the granular sludge was sampled from the bottom and the middle of the UASB reactor to evaluate the distribution of functional genes, with the samples named as a and b, respectively.

The experimental data were expressed as mean ± standard deviations. Statistical analyses were carried out using Origin v9.1.

### Biodegradability

Biodegradability can be used to analyze the AD performance of WVWW. It is calculated from the experimental methane production (EMP) and the theoretical methane production [TMP, 350 mL CH_4_/g COD (Lin et al., 1999) expressed in Li et al. (2018)] as follows:

(1) $$\mathrm{BD}=\frac{\text{EMP}}{\text{TMP}}\times 100\%$$

### Analytical Methods

#### Generated Gas Analysis

The volume of biogas collected daily was measured by a medical syringe at room temperature, and the components quantitatively analyzed by gas chromatography (GC-6890, Agilent, United States). About 0.4 mL gas sample was injected into a GC equipped with a thermal conductivity detector and a packed TDX-01 stainless-steel column, with argon, the carrier gas, flowing at 50 mL/min. The inlet temperature, detector temperature, and constant oven temperature of the GC were 120, 150, and 100°C, respectively.

#### Bacterial Functional Gene Prediction

The collected granular sludge samples were analyzed by high-throughput sequencing technology for bacterial functional genes prediction. The DNA of each microbial sample was extracted by the PowerSoil DNA Isolation Kit (Mo Bio Laboratories Inc., Carlsbad, CA, United States). The extracted genomic DNA was detected by electrophoresis on 1% agarose gel. The DNA molecules were amplified using TransStart Fastpfu DNA Polymerase (TransGen AP221-02) by a PCR Amplifier (9700, ABI GeneAmp^®^, United States). The V3 and V4 regions of the 16S rRNA genes of bacteria were amplified with (5′-ACTCCTACGGGAGGCAGCAG-3′)/806R and (5′-GGACTACHVGGGTWTCTAAT-3′) as primers. The PCR amplifying procedure involved denaturation at 94°C for 5 min followed by a total of 25 cycles, including cycles at 50 and 95°C for 30 s and 72°C for 40 s. The final extension program was performed at 72°C for 7 min.

The amplified genome was transferred to an Illumina MiSeq platform (Illumina, CA, United States) for sequencing by Majorbio Bio-Pharm Technology Co. Ltd., Shanghai, China. The samples were differentiated using barcodes and primers at the ends of sequences to obtain effective sequences. The sequence directions were corrected to produce optimized data, with all sequences classified at the 97% similarity level. Using the Silva (Release 128) database as the reference standard, the acquired sequences were taxonomically analyzed by the ribosomal database project (RDP) classifier at a confidence threshold of 0.7. The abundance-related indexes (ACE) and Chao, and diversity-related indexes, Shannon and Simpson, were calculated based on operational taxonomic units (OTUs). Rarefaction curves were produced to demonstrate the reliability of the sequencing data. The results of the high-throughput sequencing were then uploaded to the Sequence Read Archive (SRA) database of the National Center for Biotechnology Information (NCBI).

The sequencing results were evaluated by OTU cluster analysis. The functional genes of the bacterial communities were predicted by PICRUSt, with the abundance of relevant functional genes also calculated. The greengene ID corresponding to the OTU was compared using the COG and KEGG databases to obtain the COG and KEGG Ontology (KO) family information, with the abundances also recorded. The relevant information and function of the COG were analyzed through the eggnog database according to the COG code.

## Results and Discussion

### Effects of WVWW on Methanogenesis

**FIGURE 1:**
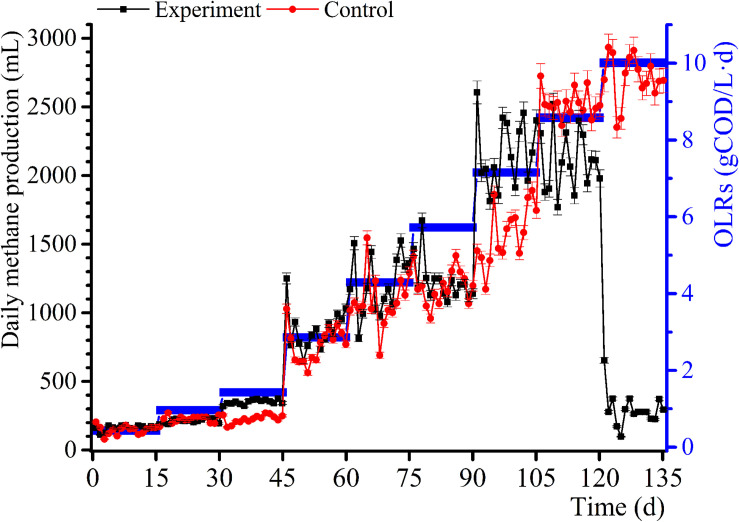
Variation in daily methane production with increasing OLRs.

**FIGURE 2:**
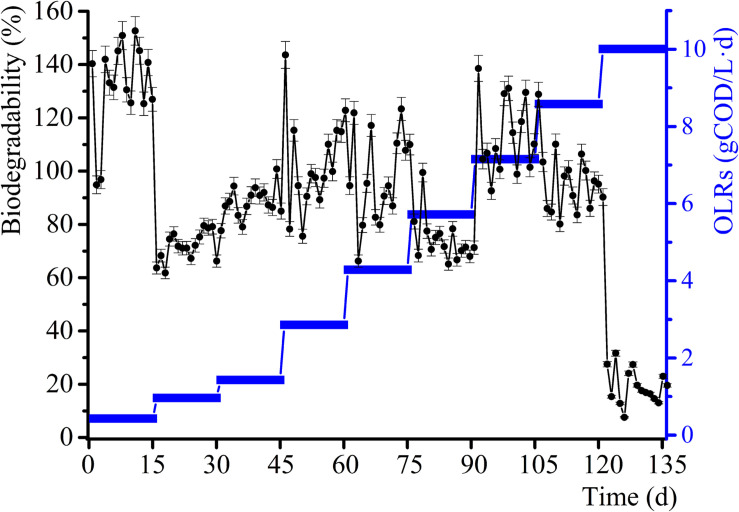
Variation in biodegradability with increasing OLRs.

The daily methane production and BD for the samples and control group experiments for diverse OLRs are displayed in Figures 1, 2. The daily methane production changes several times as the OLRs increase. At the early continuous AD (OLR ≤ 5.72 g COD/L⋅d) stage, the daily methane production values for the experimental samples are comparable to that of the control experiment. At an OLR of 7.15 g COD/L⋅d, the daily methane production of the WVWW sample is slightly higher than that of the control sample, suggesting that the essential elements in WVWW and biodegradable organic matter are conducive for methanogenesis. Zheng et al. (2016) also reported that HTLWW with a low volume ratio is non-toxic for anaerobic microorganisms, possibly enhancing small organics (organic acids, alcohols, etc.) supply and nutrients (N, P, etc.) for microbial metabolism. The trend of daily methane production then reverses; that is, results for the experimental samples are lower than that for the control sample, indicating that toxicants progressively hinder the methane production. The data for the experimental samples exhibit a cliff-like decline, mainly because high toxicants concentrations are severely inhibiting the methane production activity of the archaea (Tommaso et al., 2015). Also, the BD values in the first phase are very high (Figure 2), possibly due to residual nutrients in the granular sludge. Overall, the BD shows an upward trend as the OLRs increase from 0.97 to 7.15 g COD/L⋅d. Further, a BD value > 1 indicates that WVWW with low concentration and high organic matter likely promotes methane production (Zheng et al., 2016). At OLR > 8.58 g COD/L⋅d, the BD variation is similar to that of the daily methane production, further demonstrating the inhibitory effect of high toxicants concentration on the activity of methanogens (Chen et al., 2017a). In addition, the BD at 10.01 g COD/L⋅d sharply decrease by approximately 20%, highlighting refractory WVWW at this OLR.

### Microbial Community Analysis

#### Effects of WVWW on Abundance and Diversity of Microbial Community

The high throughput sequencing of Illumina MiSeq was employed in analyzing bacterial structure changes during continuous AD of WVWW. The indexes of phylogenetic abundance and diversity of the microbial communities are presented in Table 1. Chen Y. et al. (2019) examined the relationships between the alpha analysis indexes and the diversity and richness of microbial communities. The Chao and ACE indexes reflect the richness of the bacterial community, with higher values corresponding to higher richness. Also, the Simpson and Shannon indexes are closely related to species diversity of a bacterial community; that is, a low Simpson index and high Shannon index mean high species diversity. Therefore, the sample IC-4 showing the highest ACE (685.57) and Chao (700.32) reflects the highest bacterial abundance. This indicates that the WVWW, under the OLR condition, is conducive for growth and reproduction of bacteria. The highest OTU value (584) for the sample also supports the earlier observation. However, sample IC-7 exhibits the highest Shannon index (4.44) and the lowest Simpson index (0.25), highlighting the highest species diversity. This suggests that specific microorganisms with strong toxicity resistance are enriched during the long-term culture.

**TABLE 1:** Abundance and diversity analysis of bacterial community for clustering at 97% identity.

**Samples**	**ACE**	**Chao**	**Shannon**	**Simpson**	**OTU**
IC-1	563.09	565.63	3.90	0.065	499
IC-2	606.24	597.47	3.94	0.055	529
IC-3	649.02	632.84	3.89	0.045	572
IC-4	685.57	700.32	4.25	0.035	584
IC-5	642.70	637.69	3.98	0.048	559
IC-6	598.92	593.56	4.13	0.037	533
IC-7	636.94	658.51	4.44	0.025	564

*IC-1, IC-2, IC-3, IC-4, IC-5, IC-6, and IC-7 represent the granular samples at OLRs of 1.43, 2.86, 4.29, 5.72, 7.15, 8.58, and 10.01 g COD/L⋅d.*

Based on OTUs at 97% similarity, the rarefaction curves were produced (Supplementary Figure S1), enabling species abundance comparison and reflection on the rationality of the sequencing data of samples. The flat shapes of the rarefaction curves indicate reliability of the sequencing results.

#### Venn Diagrams Comparison

**FIGURE 3:**
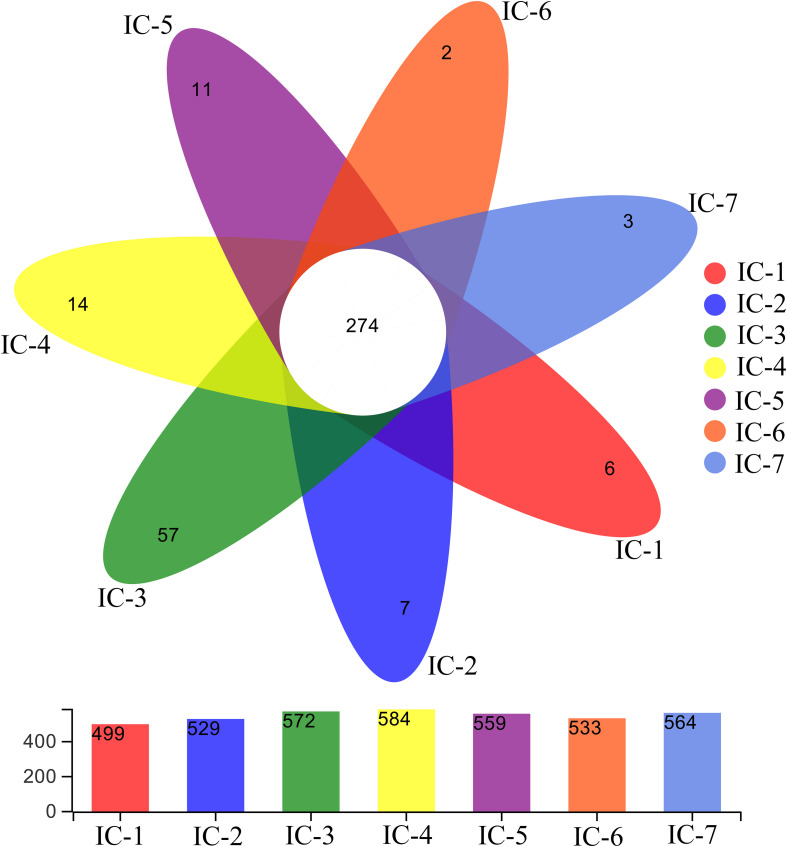
Venn diagram of samples from UASB reactor. The numbers represent OTUs at 97% similarity. IC-1, IC-2, IC-3, IC-4, IC-5, IC-6, and IC-7 represent the granular samples at OLRs of 1.43, 2.86, 4.29, 5.72, 7.15, 8.58, and 10.01 g COD/L⋅d.

A Venn diagram is used for counting the common and unique species of the seven samples (Figure 3), thereby intuitively expressing species composition and species sharing similarity for the OTU level at 97% similarity (Chen J. et al., 2019). The total OTU for the seven samples indicate significant variation with increasing OLRs (from 499 to 584). Throughout the continuous AD, the shared OTU value is 274, with the unique OTU values generally increasing initially, and then decreasing with increasing OLRs, consistent with the total OTU. This indicates that the complex organic compounds in WVWW under low OLRs promote microorganism enrichment. Meanwhile, these organisms improve methanogenesis of wastewater and degradation of organic matter. Previous studies also suggest that bacterial abundance is closely related to the degradation efficiency of organics (Zhang et al., 2019c). Nevertheless, increasing OLRs create higher concentrations of toxic substances, and the toxic effects hamper the metabolism and reproduction of common microorganisms (Zheng et al., 2016). Evidently, selective enrichment of toxicity-tolerant microorganisms occurs during long-term AD culture.

#### Microbial Community Structures

The bacterial composition of the seven samples are taxonomically analyzed at the phylum level by the Illumina Miseq sequencing based on the RDP classifier (Table 2). The long-term AD culture caused obvious evolution of the dominant bacteria comprising the phyla *Chloroflexi*, *Firmicutes*, *Proteobacteria*, *Acidobacteria*, *Synergistetes*, and *Actinobacteria*. Among these, phyla *Chloroflexi*, *Firmicutes*, *Proteobacteria*, *Synergistetes*, and *Actinobacteria* were also frequently detected in AD of HTLWW (Si et al., 2016; Chen et al., 2017b). The phyla *Firmicutes* and *Proteobacteria*, symbiotic with hydrogenotrophic methanogens, absorb carbohydrates and polycyclic aromatic hydrocarbons as carbon sources, with acetate, hydrogen, and carbon dioxide as the final products (Cheng et al., 2013; Rosenberg, 2013). *Proteobacteria* is the main bacterial phylum in a sewage treatment system (Martiny et al., 2005), whereas *Synergistetes* and *Actinobacteria* can degrade organic acids and glucose (Honda et al., 2013; Rosenberg et al., 2014a). The *Chloroflexi* degrades phenol, yielding volatile organic acids (VFAs) as metabolic products (Rosenkranz et al., 2013). With increasing OLRs, the dominant bacterial phyla abundance initially increases and then decreases. This characteristic is mainly attributed to organics at low OLRs providing abundant nutrients for growth and reproduction of microorganisms, whereas at high OLRs, the wastewater contains relatively high concentrations of toxicants that inhibit microbial activity (Zheng et al., 2016). However, the increase in the *Actinobacteria* phylum suggest some of its species are associated with detoxification.

**TABLE 2:** Relative abundances of bacteria community at phylum level through Illumina Miseq sequencing.

**Phyla**	**IC-1 (%)**	**IC-2 (%)**	**IC-3 (%)**	**IC-4 (%)**	**IC-5 (%)**	**IC-6 (%)**	**IC-7 (%)**
*Chloroflexi*	13.17	28.92	32.65	21.54	31.03	21.98	18.11
*Firmicutes*	18.71	27.45	2.22	23.46	8.30	20.11	25.5
*Proteobacteria*	28.48	7.58	4.55	7.36	6.58	5.91	8.23
*Acidobacteria*	4.53	5.06	9.26	7.34	15.16	10.85	8.13
*Synergistetes*	9.62	7.31	6.96	6.93	5.64	10.01	6.74
*Actinobacteria*	4.80	4.97	4.83	7.12	4.44	7.14	9.75
*Bacteroidetes*	7.57	6.01	3.21	3.29	2.09	2.45	3.55
*Thermotogae*	0.66	0.69	8.73	3.26	4.08	3.82	4.44
*Aegiribacteria*	1.38	1.67	6.17	2.57	5.32	3.74	3.07
*Atribacteria*	1.79	1.94	6.33	3.47	2.47	2.50	2.12

*IC-1, IC-2, IC-3, IC-4, IC-5, IC-6, and IC-7 represent the granular samples at OLRs of 1.43, 2.86, 4.29, 5.72, 7.15, 8.58, and 10.01 g COD/L⋅d.*

**FIGURE 4:**
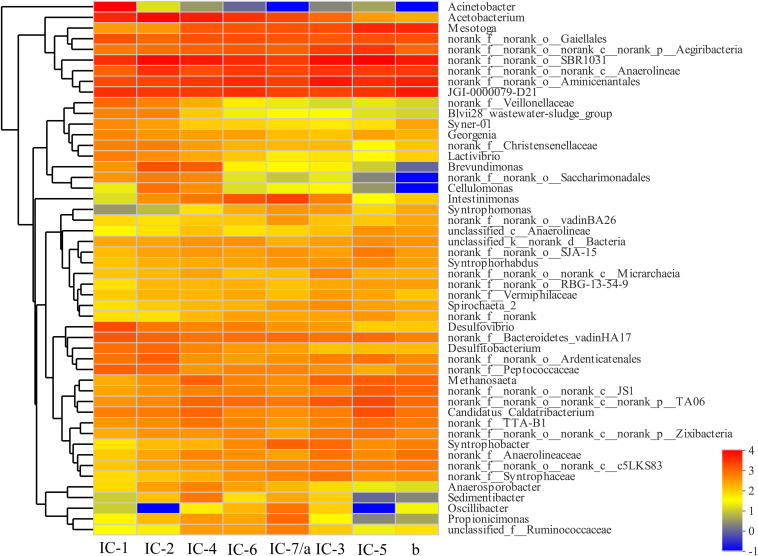
Bacterial heatmap at genus level through Illumina Miseq sequencing. IC-1, IC-2, IC-3, IC-4, IC-5, IC-6, and IC-7 represent the granular samples at OLRs of 1.43, 2.86, 4.29, 5.72, 7.15, 8.58, and 10.01 g COD/L⋅d. a and b represent sludge samples at different locations of UASB at OLR of 10.01 g COD/L⋅d.

The bacterial community composition differs significantly with increasing OLRs at the genus level based on a heatmap (Figure 4). The *Anaerolineae*, *Aminicenantales*, and *Acetobacterium* are the dominant genera in all samples. The *Anaerolineae* (*Chloroflexi* phylum) is mainly responsible for fermentation of glucose and VFAs production, which also reflects detoxication (e.g., phenol degradation) (Rosenkranz et al., 2013). Most bacteria including *Acetobacterium* (*Proteobacteria* phylum), *Desulfovibrio* (*Proteobacteria* phylum), *Peptococcaceae* (*Firmicutes* phylum), *Christensenellaceae* (*Firmicutes* phylum), *Syntrophomonae* (*Firmicutes* phylum), and *Syntrophorhabdus* (*Proteobacteria* phylum) mainly promote organics acidification. The bacterial metabolic products related to acidification are mostly organic acids, hydrogen, and carbon dioxide (Morotomi et al., 2012; Rosenberg et al., 2014b). In addition to acidification, some bacteria including *Desulfovibrio* (*Proteobacteria* phylum) and *Syntrophorhabdus* (*Proteobacteria* phylum) exhibit detoxification characteristics, promoting degradation of aromatic aldehydes and aromatic compounds such as furfural (Zhao et al., 2012; Rosenberg et al., 2014b).

### Functional Genes Comparison

#### Functional Genes Comparison at Diverse OLRS

**FIGURE 5:**
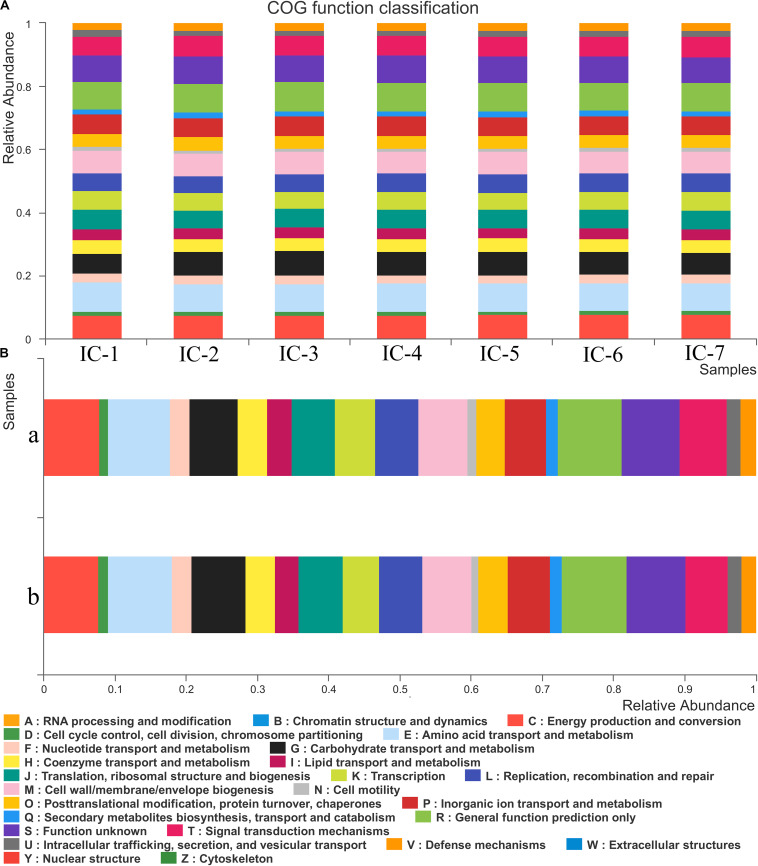
COG function classification and abundance. **(A)** Sludge samples at diverse OLRs; **(B)** Sludge samples at different locations of the UASB reactor. IC-1, IC-2, IC-3, IC-4, IC-5, IC-6, and IC-7 represent the granular samples at OLRs of 1.43, 2.86, 4.29, 5.72, 7.15, 8.58, and 10.01 g COD/L⋅d. a and b represent sludge samples at different locations of UASB at OLR of 10.01 g COD/L⋅d.

According to Figure 5A, 25 COG functional genes related to RNA processing and modification, energy production and conversion, carbohydrate transport, and metabolism, and coenzyme transport and metabolism are detected. The seven samples are in similar COG functional gene classification, but their relative abundance change significantly with increasing OLRs. The gene abundance related to energy production and conversion is relatively high, such as transport and metabolism of amino acid, coenzyme, and lipid (Figure 5A). These are mainly responsible for carbon forms conversion, playing an essential role in the methanogenic process during AD. The metabolism of coenzyme and amino acid can synthesize protein and polypeptide, which are essential for the growth and reproduction of microorganism. However, lipid was not detected in the wastewater, and genes associated with lipid metabolism may be responsible for cell membrane structure. Genes associated with carbohydrate transport and metabolism can promote carbohydrate degradation (Liu et al., 2019). The trace carbohydrate in WVWW may account for the scarcity of related functional genes. The low genes linked to defense mechanisms also demonstrate that anaerobic microorganisms are susceptible to toxicants, which is consistent with the observation that high OLRs reflect low methane production (Figure 1). Cell growth and reproduction are primarily related to protein turnover, replication, and recombination and repair genes.

#### Functional Genes Comparison at Different UASB Locations

From Figure 5B, the distribution of genes along the longitudinal direction of the UASB reactor look similar. Moreover, few differences exist in the species of related functional genes, indicating that the functional microorganisms are homogeneous. However, the functional genes abundance in the middle of the UASB reactor is slightly higher than that at the bottom, suggesting that the concentration of toxic substances decreases in the longitudinal direction of the reactor. Functional analyses of predictive genes also support this viewpoint such as amino acid transport and metabolism, carbohydrate transport and metabolism, inorganic ion transport, and metabolism, and general function prediction. And the decreased abundance of bacteria such as *Anaerolineae*, *Aminicenantales*, etc., which are associated with the detoxification of wood vinegar, also indicated lower toxicity in the middle of UASB (Figure 4). Low toxicity can accelerate the transformation and metabolism of inorganic ions, amino acids, and other small molecules. The bacteria at the bottom of the UASB reactor are mainly related to the acidification and detoxification of organisms, which is consistent with the higher abundance of genes related to defense mechanisms in the microbial samples at the bottom of the reactor than in the middle.

## Conclusion

In this study, we investigated methane production, microbial communities, and functional genes prediction associated with continuous AD of WVWW. When the OLR was >8.58 g COD/L⋅d, the AD performance deterioration was mainly manifested by decreased daily methane production and BD. The common dominant bacteria for the seven samples at the phylum level were *Chloroflexi*, *Firmicutes*, *Proteobacteria*, *Acidobacteria*, *Synergistetes*, and *Actinobacteria*, and these functioned mainly in the acidification and detoxification of substrates. However, the structure and abundance of bacteria significantly changed at the genus level with increasing OLRs, suggesting that long-term continuous AD can promote the selective enrichment of functional bacteria. The functional genes associated with energy production and conversion, amino acid transport and metabolism, carbohydrate transport and metabolism, and inorganic ion transport and metabolism were predicted to dominate. Predicting genes functions provides a basis for further screening of functional genes.

## Data Availability Statement

All datasets presented in this study are included in the article/Supplementary Material.

## Author Contributions

DH and QF performed the experiments and wrote the manuscript. YZ and HX designed the research. LC and HS analyzed the data. YL analyzed the data and reviewed the manuscript. All authors read and approved the final manuscript.

## Conflict of Interest

The authors declare that the research was conducted in the absence of any commercial or financial relationships that could be construed as a potential conflict of interest.

## Abbreviations

ACE: abundance-related indexes
AD: anaerobic digestion
BD: biodegradability
COD: chemical oxygen demand
EMP: experimental methane production
GC: gas chromatography
HRT: hydraulic retention time
HTLWW: hydrothermal liquefaction wastewater
KO: KEGG ontology
OLRs: organic loading rates
OTUs: operational taxonomic units
RDP: ribosomal database project
SRA: sequence read archive
TMP: theoretical methane production
TS: total solids
UASB: up-flow anaerobic sludge bed
VFAs: volatile organic acids
VS: volatile solids
WVWW: wood vinegar wastewater.

## References

[B1] Chen H. Zhang C. Rao Y. Jing Y. Gang L. (2017a). Methane potentials of wastewater generated from hydrothermal liquefaction of rice straw: focusing on the wastewater characteristics and microbial community compositions. *Biotechnol. Biofuels* 10 1–16. 28580014 10.1186/s13068-017-0830-0 PMC5452606

[B2] Chen H. Zhang C. Rao Y. Jing Y. Luo G. Zhang S. (2017b). Methane potentials of wastewater generated from hydrothermal liquefaction of rice straw: focusing on the wastewater characteristics and microbial community compositions. *Biotechnol. Biofuels* 10:140. 10.1186/s13068-017-0830-0 PMC5452606 28580014

[B3] Chen J. Yang Y. Liu Y. Tang M. Wang R. Zhang C. (2019). Bacterial community shift in response to a deep municipal tail wastewater treatment system. *Bioresour. Technol.* 281 195–201. 10.1016/j.biortech.2019.02.099 30822640

[B4] Chen Y. Lin T. Chen W. (2019). Enhanced removal of organic matter and typical disinfection byproduct precursors in combined iron–carbon micro electrolysis-UBAF process for drinking water pre-treatment. *J. Environ. Sci.* 78 315–327. 10.1016/j.jes.2018.11.010 30665651

[B5] Cheng H. Whang L. Lin C. Liu C. Wu C. (2013). Metabolic flux network analysis of fermentative hydrogen production: using *Clostridium tyrobutyricum* as an example. *Bioresour. Technol.* 141 233–239. 10.1016/j.biortech.2013.03.141 23659760

[B6] Fan Q. Li Y. Zhao Y. Xu H. Chen L. Hua D. (2020). Anaerobic digestion coupled with three-dimensional iron-carbon electrolysis for enhanced treatment of wood-vinegar wastewater and bacterial structure changes. *J. Clean. Prod.* 267:122095 10.1016/j.jclepro.2020.122095

[B7] Gu Y. Zhang X. Deal B. Han L. (2019). Biological systems for treatment and valorization of wastewater generated from hydrothermal liquefaction of biomass and systems thinking: a review. *Bioresour. Technol.* 278 329–345. 10.1016/j.biortech.2019.01.127 30723025

[B8] Honda T. Fujita T. Tonouchi A. (2013). *Aminivibrio pyruvatiphilus* gen. nov., sp. nov., an anaerobic, amino-acid-degrading bacterium from soil of a Japanese rice field. *Int. J. Syst. Evol. Microbiol.* 63(Pt 10), 3679–3686. 10.1099/ijs.0.052225-0 23625260

[B9] Li W. Khalid H. Zhu Z. Zhang R. Liu G. Chen C. (2018). Methane production through anaerobic digestion: participation and digestion characteristics of cellulose, hemicellulose and lignin. *Appl. Energy* 226 1219–1228. 10.1016/j.apenergy.2018.05.055

[B10] Lin J. Ma Y. Chao A. C. Huang C. (1999). BMP test on chemically pretreated sludge. *Bioresour. Technol.* 68 187–192. 10.1016/s0960-8524(98)00126-6

[B11] Liu D. Xie B. Dong Y. Liu H. (2019). Semi-continuous fermentation of solid waste in closed artificial ecosystem: microbial diversity, function genes evaluation. *Life Sci. Space Res.* 25 136–142. 10.1016/j.lssr.2019.10.003 32414487

[B12] Liu L. Guo X. Wang S. Li L. Zeng Y. Liu G. (2018). Effects of wood vinegar on properties and mechanism of heavy metal competitive adsorption on secondary fermentation based composts. *Ecotoxicol. Environ. Saf.* 150 270–279. 10.1016/j.ecoenv.2017.12.037 29289862

[B13] Martiny A. C. Albrechtsen H. J. Arvin E. Molin S. (2005). Identification of bacteria in biofilm and bulk water samples from a nonchlorinated model drinking water distribution system: detection of a large nitrite-oxidizing population associated with *Nitrospira* spp. *Appl. Environ. Microbiol.* 71 8611–8617. 10.1128/aem.71.12.8611-8617.2005 16332854 PMC1317318

[B14] Morotomi M. Nagai F. Watanabe Y. (2012). Description of *Christensenella minuta* gen. nov., sp. nov., isolated from human faeces, which forms a distinct branch in the order Clostridiales, and proposal of Christensenellaceae fam. nov. *Int. J. Syst. Evol. Microbiol.* 62 144–149. 10.1099/ijs.0.026989-0 21357455

[B15] Pimenta A. S. Fasciotti M. Monteiro T. V. C. Lima K. M. G. (2018). Chemical composition of pyroligneous acid obtained from eucalyptus GG100 clone. *Molecules* 23:426. 10.3390/molecules23020426 29462854 PMC6017387

[B16] Quispe-Arpasi D. de Souza R. Stablein M. Liu Z. Duan N. Lu H. (2018). Anaerobic and photocatalytic treatments of post-hydrothermal liquefaction wastewater using H2O2. *Bioresour. Technol. Rep.* 3 247–255. 10.1016/j.biteb.2018.08.003

[B17] Rosenberg E. (2013). *The Prokaryotes: Alphaproteobacteria and Betaproteobacteria.* Cham: Springer.

[B18] Rosenberg E. DeLong E. F. Lory S. Stackebrandt E. Thompson F. (2014a). *The Prokaryotes: Actinobacteria.* Cham: Springer.

[B19] Rosenberg E. Long E. F. Loy S. Stackebrandt E. Thompson F. (2014b). *The Prokaryotes: Deltaproteobacteria and Epsilonproteobacteria.* Cham: Springer.

[B20] Rosenkranz F. Cdbrol L. Carballa M. Donoso-Bravo A. Cruz L. Ruiz-Filippi G. (2013). Relationship between phenol degradation efficiency and microbial community structure in an anaerobic SBR. *Water Res.* 47 6739–6749. 10.1016/j.watres.2013.09.004 24083853

[B21] Shen Y. Zhang N. Zhang S. (2020). Catalytic pyrolysis of biomass with potassium compounds for Co-production of high-quality biofuels and porous carbons. *Energy* 190:116431 10.1016/j.energy.2019.116431

[B22] Si B. Li J. Zhu Z. Shen M. Lu J. Duan N. (2018). Inhibitors degradation and microbial response during continuous anaerobic conversion of hydrothermal liquefaction wastewater. *Sci. Total Environ.* 630 1124–1132. 10.1016/j.scitotenv.2018.02.310 29554734

[B23] Si B. Li J. Zhu Z. Zhang Y. Lu J. Shen R. (2016). Continuous production of biohythane from hydrothermal liquefied cornstalk biomass via two-stage high-rate anaerobic reactors. *Biotechnol. Biofuels* 9:254. 10.1186/s13068-016-0666-z PMC5117538 27895708

[B24] Theapparat Y. Chandumpai A. Leelasuphakul W. Laemsak N. (2015). Pyroligneous acids from carbonisation of wood and bamboo: their components and antifungal activity. *J. Trop. For. Sci.* 27 517–526.

[B25] Tommaso G. Chen W. Li P. Schideman L. Zhang Y. (2015). Chemical characterization and anaerobic biodegradability of hydrothermal liquefaction aqueous products from mixed-culture wastewater algae. *Bioresour. Technol.* 178 139–146. 10.1016/j.biortech.2014.10.011 25455086

[B26] Usman M. Hao S. Chen H. Ren S. Tsang D. C. W. O-Thong S. (2019). Molecular and microbial insights towards understanding the anaerobic digestion of the wastewater from hydrothermal liquefaction of sewage sludge facilitated by granular activated carbon (GAC). *Environ. Int.* 133(Pt B), 105257. 10.1016/j.envint.2019.105257 31675572

[B27] Wirth B. Krebs M. Andert J. (2015). Anaerobic degradation of increased phenol concentrations in batch assays. *Environ. Sci. Pollut. Res.* 22 19048–19059. 10.1007/s11356-015-5100-8 26233745

[B28] Yang L. Si B. Martins M. A. Watson J. Chu H. Zhang Y. (2018). Improve the biodegradability of post-hydrothermal liquefaction wastewater with ozone: conversion of phenols and N-heterocyclic compounds. *Water Sci. Technol.* 2017 248–255. 10.2166/wst.2018.108 29698239

[B29] Zhang F. Shao J. Yang H. Guo D. Chen Z. Zhang S. (2019a). Effects of biomass pyrolysis derived wood vinegar on microbial activity and communities of activated sludge. *Bioresour. Technol.* 279 252–261. 10.1016/j.biortech.2019.01.133 30735935

[B30] Zhang F. Yang H. Guo D. Zhang S. Chen H. Shao J. A. (2019b). Effects of biomass pyrolysis derived wood vinegar (WVG) on extracellular polymeric substances and performances of activated sludge. *Bioresour. Technol.* 274 25–32. 10.1016/j.biortech.2018.11.064 30500760

[B31] Zhang W. Ren X. He J. Zhang Q. Qiu C. Fan B. (2019c). Application of natural mixed bacteria immobilized carriers to different kinds of organic wastewater treatment and microbial community comparison. *J. Hazard. Mater.* 377 113–123. 10.1016/j.jhazmat.2019.05.068 31154198

[B32] Zhao C. Gao Z. Qin Q. Li F. Ruan L. (2012). *Desulfobaculum xiamenensis* gen. nov., sp. nov., a member of the family Desulfovibrionaceae isolated from marine mangrove sediment. *Int. J. Syst. Evol. Microbiol.* 62(Pt 7), 1570–1575. 10.1099/ijs.0.036632-0 21873514

[B33] Zheng M. Schideman L. C. Tommaso G. Chen W. Zhou Y. Nair K. (2016). Anaerobic digestion of wastewater generated from the hydrothermal liquefaction of Spirulina: toxicity assessment and minimization. *Energy Conv. Manag.* 141 420–428. 10.1016/j.enconman.2016.10.034

[B34] Zhu X. Li Y. Wang X. (2019). Machine learning prediction of biochar yield and carbon contents in biochar based on biomass characteristics and pyrolysis conditions. *Bioresour. Technol.* 288:121527. 10.1016/j.biortech.2019.121527 31136889

