# Supplementary material for: Continuous Anaerobic Digestion of Wood Vinegar Wastewater From Pyrolysis: Microbial Diversity and Functional Genes Prediction
Source: Front Bioeng Biotechnol. 2020 Aug 5;8:923. doi: 10.3389/fbioe.2020.00923 (PMC7422680; doi:10.3389/fbioe.2020.00923)
Supplement: Supplementary file 1 [file Data_Sheet_1.docx]

Supplementary Materials

**Continuous anaerobic digestion of wood vinegar wastewater from pyrolysis: microbial diversity and functional genes prediction**

Dongliang Hua^1†^, Qingwen Fan^1†^, Yuxiao Zhao^1^, Haipeng Xu^1^, Lei Chen^1^, Hongyu Si^1^, Yan Li^1,2^ *

^1^ Energy Research Institute, Qilu University of Technology (Shandong Academy of

Sciences), Shandong Provincial Key Laboratory of Biomass Gasification Technology,

Jinan 250014, China;

^2^ State Key Laboratory of Microbial Technology, Shandong University, Qingdao

266237, China;

† These authors contributed equally to this work.

*Corresponding author: Yan Li*, E-mail: [liy@sderi.cn](mailto:liy@sderi.cn)

Present affiliation: Energy Research Institute, Qilu University of Technology (Shandong Academy of Sciences), Shandong Provincial Key Laboratory of Biomass Gasification Technology, Jinan 250014, China


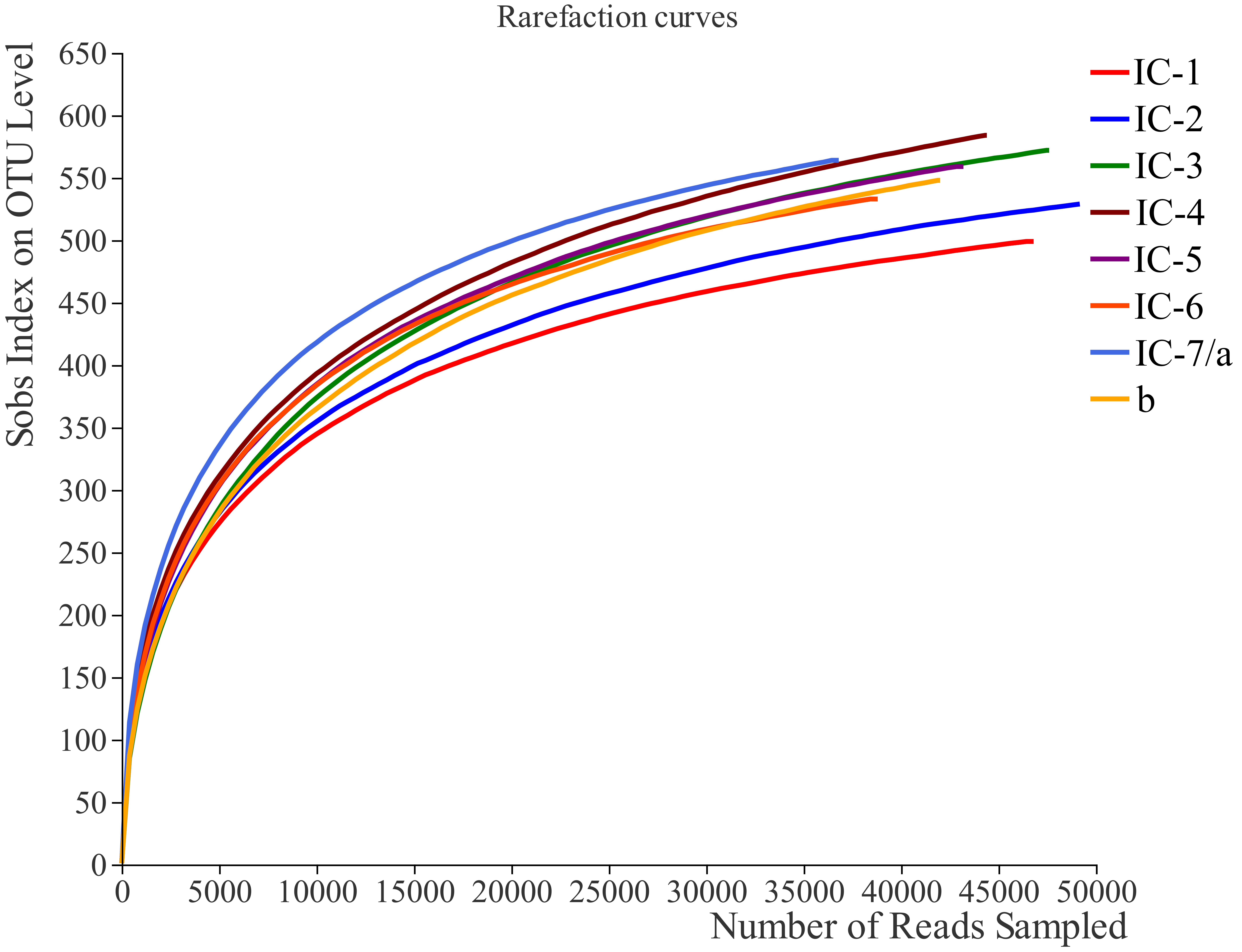


**Figure S1.** Rarefaction curves based on the 16S rRNA gene sequencing of samples.

IC-1, IC-2, IC-3, IC-4, IC-5, IC-6, and IC-7 represent the granular samples at OLRs of 1.43, 2.86, 4.29, 5.72, 7.15, 8.58, and 10.01 g COD/L·d. a and b represent sludge samples at different locations of UASB at OLR of 10.01 g COD/L·d.
